# Nutrient quality and maturity status of frass fertilizer from nine edible insects

**DOI:** 10.1038/s41598-022-11336-z

**Published:** 2022-05-03

**Authors:** Dennis Beesigamukama, Sevgan Subramanian, Chrysantus M. Tanga

**Affiliations:** 1 International Centre of Insect Physiology and Ecology, P.O. Box 30772-00100, Nairobi, Kenya; 2 Department of Crop Production and Management, Busitema University, P.O. Box 236, Tororo, Uganda

**Keywords:** Biogeochemistry, Environmental sciences

## Abstract

Globally, there is growing interest to recycle organic waste using insect larvae into high-quality frass fertilizer through circular economy approach. This paper presents the first comparative report on the nutrient concentrations, fertilizing indices, nutrient supply potentials and compost maturity of nine edible insect frass fertilizers. Our results revealed that frass fertilizers from all the insect species had adequate concentrations and contents of macronutrients [nitrogen (N), phosphorus (P), potassium (K)], secondary nutrients (calcium, magnesium, and sulphur) and micro-nutrients (manganese, copper, iron, zinc, boron, and sodium). The fertilizing indices of the frass fertilizers were above 3. However, black soldier fly (BSF) frass fertilizer had significantly higher N (20–130%) and K (17–193%) concentrations compared to others. The P concentration of *Gryllus bimaculatus* frass fertilizer was 3–800% higher compared to those of frass fertilizers from other insect species. The potential N and K supply capacities of BSF frass fertilizer was 19–78% and 16–190% higher, respectively. The P supply capacity of cricket frass fertilizer was 17–802% higher compared to others. The highest seed gemination rate (> 90%) and germination index (267%) were observed in seeds treated with BSF frass fertilizer. Frass fertilizer obtained from the other eight insect species showed medium to high phytotoxicity. These findings demonstrate that insect frass fertilizers are promising alternatives to existing commercial fertilizers (i.e., mineral, and organic) for improved soil health and crop yield.

## Introduction

Soil degradation and poor waste management are major challenges to environmental health, and food and nutrition security in sub-Saharan Africa (SSA)^1–7^. About 40% of soils in SSA are deficient in most nutrients required for crop growth, with 25% affected by aluminium toxicity, 18% prone to leaching and 8.5% characterized by phosphorus fixation^8^. Despite the challenges, most smallholder farmers use little (≤ 10 kg ha^−1^ year^−1^) or no mineral fertilizer due to the high-cost implications and limited access^3^. Even in situations where mineral fertilizers are widely used, their efficiency is hindered by low soil organic matter, micronutrient deficiencies and high soil acidity^9–12^. Although the use of organic fertilizer is acceptable and affordable to farmers^13–16^, there has been limited uptake in SSA due to poor quality, long production time as well as limited sources of organic matter on the farm^10,17,18^. Thus, there is need to explore alternative sources of organic fertilizers that are readily available, affordable and of good quality such as insect frass fertilizer.

The use of insects as bio-converters of low-value organic matter into affordable and high-quality food, feed, fibre and organic fertilizer products has rapidly attracted attention globally^19–26^. Several insect species are being mass produced at the International Centre of Insect Physiology and Ecology (*icipe*) including black soldier fly (BSF) (*Hermertia illucens* L.), two-spotted crickets (*Gryllus bimaculatus* De Geer) and *Scapsipedus icipe* Hugel and Tanga), silk moth (*Bombyx mori* L.), edible saturniid caterpillar [*Gonimbrasia krucki* Nudaurelia), mealworm (*Tenebrio molitor* L.), desert locust (*Schistocerca gregaria* Forsskål), African fruit beetle (*Pachnoda sinuata* L.) and rhinoceros beetle (*Oryctes rhinoceros* L.). Among these insects, only the frass fertilizer from *H. illucens* and *T. molitor* have been tested and proven to play a critical role in improving soil fertility, yield and the nutritional quality of different crops^27–33^.

Insect mass rearing using organic waste could contribute to addressing the challenges of poor waste management and low soil fertility in SSA^2,4,6,34,35^. Insect-mediated bioconversion of organic waste into organic fertilizers could reduce on land filling and return nutrients to agricultural lands. The bioconversion of organic waste into high value commercial products is a positive step towards sustainable waste recycling, whereby the income and other nonmonetary benefits obtained could act as incentives towards improved waste management and circular economy^36,37^. For example, it has been demonstrated that *H. illucens* larvae require only 5 weeks to recycle organic wastes into nutrient-rich mature and stable frass fertilizer compared to 8–24 weeks for conventional composting^26^. In Kenya, ex-ante macroeconomic estimates revealed that adoption of insect bioconversion technology can recycle between 2 and 18 million tonnes of waste into organic fertilizer worth approximately 9–85 million USD/year^38^.

Studies on the utilization of frass fertilizer from edible and commercial insects as organic fertilizer are still limited, except for *H. illucens* and *T. molitor*^26,30,33,39–42^. There is inadequate research on the fertilizer quality of frass fertilizer generated by other insect species. There is urgent need to explore the nutrient quality of different frass fertilizer products to ensure future diversification of suitable organic fertilizer products in the market that can replace or serve as important alternatives to the low quality organic fertilizers used in most African cropping systems^15–17,43,44^. The nutrient quality and fertilizing index of frass fertilizer generated by most insects are largely unknown, yet such information would guide recommendations for field application as organic fertilizers^45^.

The scanty research on the maturity and stability status of frass fertilizers obtained from most edible insects makes it difficult to determine whether they are mature or stable for field application^46,47^. Previous studies have shown that the application of immature and unstable compost causes nutrient immobilization and phytotoxicity which reduce seed germination, crop growth and yield^48–51^. This study hypothesized that the nutrient quality of frass fertilizer derived from different edible insect species are highly suitable as organic fertilizer compared to that of black soldier fly and mealworm, which have received adequate research attention and global acceptance as alternative fertilizers for soil amendment and crop production. Therefore, the current study was conceptualized to comparatively establish the nutrient concentrations, potential nutrient supply, fertilizing indices, maturity and stability of frass fertilizers generated from nine different insect species that are mass produced at *icipe*. This information would be a prerequisite to inform policy makers to develop and promote guidelines for their integration into already existing agro-input markets (i.e., fertilizer) and farming practices.

## Results

### Moisture, total organic carbon, and mineral nitrogen levels of edible insect frass fertilizer

The moisture content of frass fertilizer produced by the different insect species varied significantly (*p* ≤ 0.001) (Table 1). The moisture content ranged between 8 and 11% whereby, the *O. rhinoceros* and *H. illucens* had the lowest and highest values, respectively. Frass fertilizer from *H. illucens*, *B. mori*, and *P. sinuata* had significantly (*p* ≤ 0.001) higher moisture content than the frass fertilizer produced by other insects. The moisture content of frass fertilizer produced by *T. molitor* and *S. icipe* was significantly (*p* ≤ 0.001) higher than those of the rest, except *H. illucens*, *B. mori* and *P. sinuata*. Likewise, frass fertilizer from *G. krucki* and *S. gregaria* had significantly (*p* ≤ 0.001) higher moisture content than the frass fertilizer produced by *O. rhinoceros* (Table 1).

**Table 1:** Selected characteristics of frass fertilizer produced by different edible insect species.

Source of frass fertilizer	Moisture (%)	Total organic carbon (%)	Ammonium	Nitrates
			(mg kg^−1^)	
*Hermertia illucens*	11.2 ± 0.31a	39.1 ± 0.12abc	30.3 ± 4.03ab	7.7 ± 4.01c
*Tenebrio molitor*	10.2 ± 0.12b	49.6 ± 0.11a	0.01 ± 0.00c	1.6 ± 0.07c
*Scapsipedus icipe*	10.1 ± 0.09b	42.0 ± 0.47ab	0.01 ± 0.00c	1.9 ± 0.19c
*Bombyx mori*	11.1 ± 0.03a	44.1 ± 10.3ab	0.01 ± 0.00c	0.01 ± 0.00c
*Gryllus bimaculatus*	8.1 ± 0.07de	42.7 ± 0.31ab	0.01 ± 0.00c	0.9 ± 0.09c
*Gonimbrasia krucki*	9.3 ± 0.09c	49.0 ± 0.29a	0.80 ± 0.10bc	0.01 ± 0.00c
*Pachnoda sinuata*	11.0 ± 0.13a	31.0 ± 0.32bc	56.1 ± 6.56a	35.3 ± 1.88b
*Schistocerca gregaria*	8.6 ± 0.37cd	40.3 ± 1.25ab	0.01 ± 0.00c	4.4 ± 0.99c
*Oryctes rhinoceros*	7.6 ± 0.06e	24.1 ± 0.75c	55.3 ± 19.0a	361.7 ± 12.2a
χ^2^-*value*	440.3	44.5	104.3	6034.5
*df*	8	8	8	8
*p* value	≤ 0.001	≤ 0.001	≤ 0.001	≤ 0.001

In the same column, means (± standard error) followed by the same letters are not significantly different at *p* ≤ 0.05, n = 3.

The concentration of total organic carbon was found to vary significantly (*p* ≤ 0.001) (Table 1). The total organic carbon concentration of the frass fertilizer samples was 24–50%, *T. molitor* and *O. rhinoceros* frass fertilizer had the lowest and highest values, respectively. *Tenebrio molitor* and *G. krucki* frass fertilizer had significantly (*p* ≤ 0.001) higher total organic carbon concentrations than the frass fertilizer produced by *P. sinuata* and *O. rhinoceros*.

There were significant (*p* ≤ 0.001) differences in the concentrations of ammonium and nitrates in frass fertilizer produced by different insects (Table 1). The ammonium concentration of the frass fertilizer samples ranged between 0.01 and 174 mg kg^−1^. The highest ammonium concentration was recorded in frass fertilizer produced by *P. sinuata*, and this was 1–5610 times higher than those of other treatments. *Pachnoda sinuata* and *O. rhinoceros* produced frass fertilizer with significantly (*p* ≤ 0.001) higher ammonium concentration than other insects, except *H. illucens*. Also, the ammonium concentration of *H. illucens* frass fertilizer was significantly (*p* ≤ 0.001) higher than those of frass fertilizer from *T. molitor*, *S. icipe*, *G. bimaculatus*, *B. mori* and *S. gregaria* by 3030 times.

Frass fertilizer from *G. crucki* and *B. mori* had the lowest nitrate concentration, while the *O. rhinoceros* produced frass fertilizer with the highest nitrate concentration, which was 10–36,170 times significantly (*p* ≤ 0.001) higher than those of frass fertilizer generated by other insects. Also, the nitrate concentration of *P. sinuata* frass fertilizer was significantly (*p* ≤ 0.001) higher than those of frass fertilizer from other insects by 4.6–3530 times, except *O. rhinoceros* frass fertilizer.

### Concentrations of macro and secondary nutrients in frass fertilizer produced by different insect species

**Figure 1:**
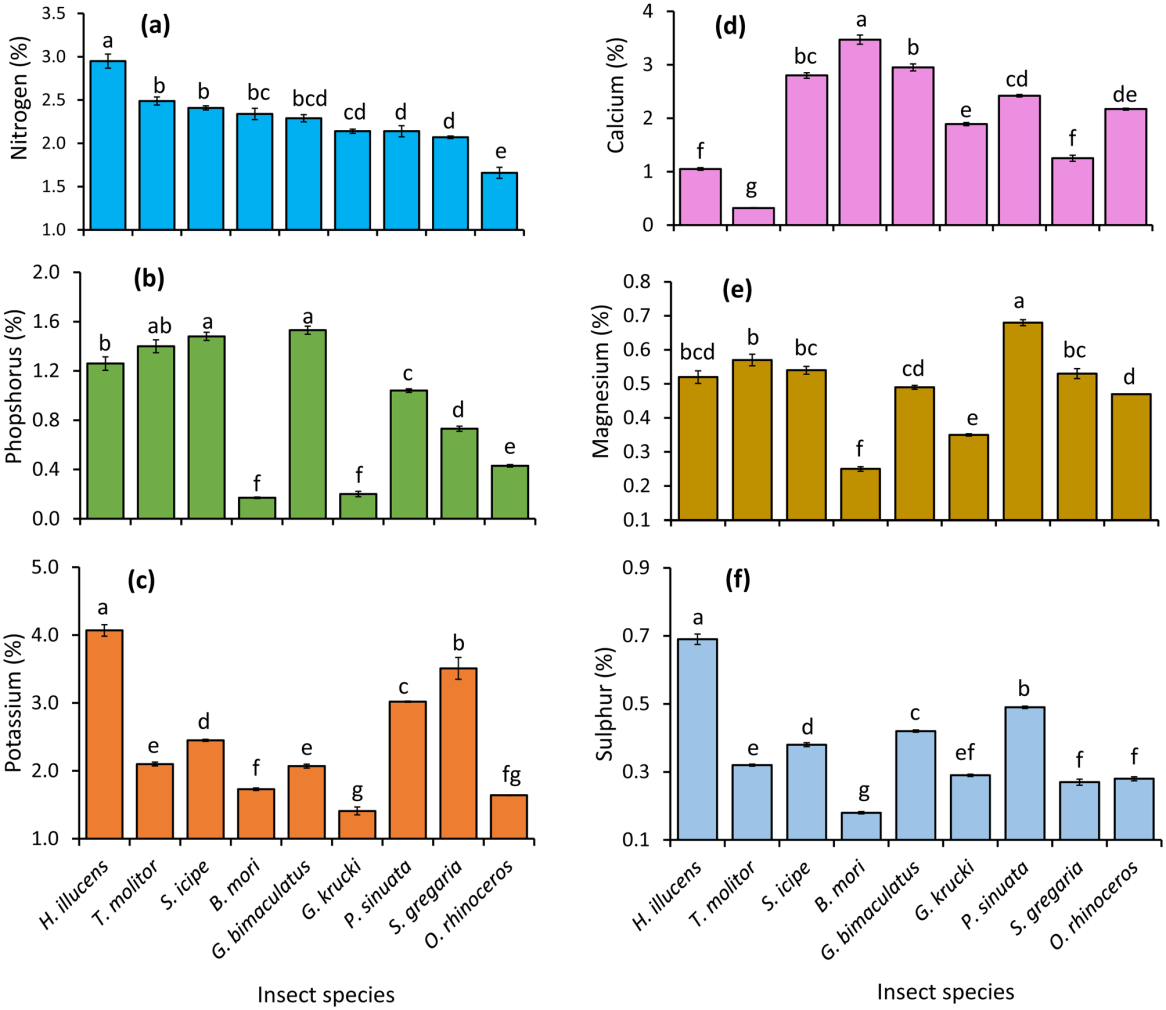
Total concentrations of nitrogen (**a**), phosphorus (**b**), potassium (**c**), calcium (**d**), magnesium (**e**) and sulphur (**f**) in frass fertilizer generated by different edible insects. Per panel, means (± standard error) followed by the same letters are not significantly different at *p* ≤ 0.05, n = 3.

There were significant differences (*p* ≤ 0.001) in the concentration of total N in frass fertilizer produced by different insects (Fig. 1a). The highest total N concentration was recorded in frass fertilizer produced by the *H. illucens*, 20–130% significantly (*p* ≤ 0.001) higher than those of frass fertilizer produced by other insects. The total N of frass fertilizer produced by *T. molitor* and *S. icipe* was significantly (*p* ≤ 0.001) higher than those of *G. krucki*, *P. sinuata*, *S. gregaria*, *O. rhinoceros*. Also, the total N concentration of frass fertilizer produced by *B. mori* was significantly (*p* ≤ 0.001) higher than those of *P. sinuata*, *S. gregaria* and *O. rhinoceros* frass fertilizer by 10, 11 and 35%, respectively. *Oryctes rhinoceros* produced frass fertilizer with the lowest total N concentration, which was significantly lower than those of frass fertilizer generated by other insects by 25–130%. Apart from *O. rhinoceros* frass fertilizer, it was noted that frass fertilizer produced by other insects would supply more than 100 kg N ha^−1^ per season, if applied for crop production at the rate of 5 t ha^−1^ (Table 3).

The concentration of total P in frass fertilizer produced by different insects was found to vary significantly (*p* ≤ 0.001) (Fig. 1b). The total P concentration ranged between 0.17 and 1.5%, whereby *G. bimaculatus* and *B. mori* produced frass fertilizer with highest and lowest P concentration, respectively. The total P concentration of frass fertilizer produced by *G. bimaculatus* and *S. icipe* was significantly (*p* ≤ 0.001) higher than those of frass fertilizer produced by other insects, except *T. molitor*. *Hermertia illucens* and *T. molitor* frass fertilizer had significantly (*p* ≤ 0.001) higher P concentration than other insect frass fertilizer samples, except *G. bimaculatus* and *S. icipe*. The P concentration of *P. sinuata* frass fertilizer was significantly (*p* ≤ 0.001) higher than frass fertilizer from *S. gregaria*, *O. rhinoceros*, *G. krucki* and *B. mori* by 1.4, 2.4, 5.2 and 6.1 folds, respectively. Also, *S. gregaria* frass fertilizer had significantly (*p* ≤ 0.001) higher P concentration when compared to those of *O. rhinoceros*, *B. mori*, and *G. krucki*. The P concentration of frass fertilizer produced by *O. rhinoceros* was 2 and 2.5 folds higher than those of frass fertilizer from *G. krucki* and *B. mori*, respectively. If applied for crop production, the frass fertilizer generated by other insects would supply 22–77 kg P ha^−1^ per season, except frass fertilizer from *B. mori* and *G. krucki* (Table 3).

There were significant differences in the concentration of total K in frass fertilizer produced by different insect species (Fig. 1c). The concentration of K in frass fertilizer produced by *H. illucens* was 17–193% higher (*p* ≤ 0.001) than those of frass fertilizer produced by other insects. The concentrations of K in frass fertilizer produced by *S. gregaria*, *P. sinuata*, and *S. icipe* were significantly (*p* ≤ 0.001) higher than those of frass fertilizer produced by other insects, except *H. illucens*. Also, the *T. molitor* and *G. bimaculatus* frass fertilizer had significantly (*p* ≤ 0.001) higher K concentration than those of frass fertilizer from *B. mori*, *O. rhinoceros* and *G. krucki*. The *B. mori* achieved significantly (*p* ≤ 0.001) higher frass fertilizer K concentration than *G. krucki* whose frass fertilizer had the lowest K concentration. Beside frass fertilizer obtained from *B. mori*, *G. krucki* and *O. rhinoceros* which contained less than 90 kg K ha^−1^ in every five tonnes of biomass*,* the frass fertilizer produced by other insects would supply between 104 and 204 kg K ha^−1^ per season if applied for crop production (Table 3).

The concentration of total Ca in frass fertilizer samples produced by different insects varied significantly (*p* ≤ 0.001) (Fig. 1d). The concentration of Ca ranged between 0.3 and 3.5%; *B. mori* and *T. molitor* frass fertilizer had the highest and lowest Ca concentrations, respectively. The Ca concentration of *T. molitor* frass fertilizer was significantly (*p* ≤ 0.001) higher than those of other insect frass fertilizer samples by 1.2–12 folds. Also*, G. bimaculatus, S. icipe* and *P. sinuata* achieved significantly (*p* ≤ 0.001) higher frass fertilizer Ca concentration than other insects, except *T. molitor* and *O. rhinoceros*. The concentrations of Ca in frass fertilizer produced by *P. sinuata*, *O. rhinoceros* and *G. krucki* were significantly (*p* ≤ 0.001) higher than those of frass fertilizer from *S. gregaria*, *H. illucens* and *T. molitor*. At the application rate of 5 t ha^−1^, frass fertilizer from all insects would supply 16–174 kg Ca ha^−1^. The Ca content of frass fertilizer from *T. molitor* was exceptionally low (Table 3).

The concentration of Mg in frass fertilizer produced by different insects also varied significantly (*p* ≤ 0.001) (Fig. 1e). The *P. sinuata* produced frass fertilizer with the highest Mg concentration, which was significantly (*p* ≤ 0.001) higher than those of other insect frass fertilizer samples by 1.2–2.7 folds. The Mg concentration of frass fertilizer produced by *T. molitor* frass fertilizer was significantly (*p* ≤ 0.001) higher than those of frass fertilizer from *G. bimaculatus*, *O. rhinoceros*, *G. krucki* and *B. mori* by 16, 21, 63 and 128%, respectively. Likewise, the Mg concentrations of frass fertilizer produced by *S. gregaria* and S. *icipe* were significantly (*p* ≤ 0.001) higher than those of frass fertilizer from *O. rhinoceros*, *G. krucki* and *B. mori* by 13–15%, 51–54% and 112–116%, respectively. The Mg concentration of frass fertilizer from *O. rhinoceros* was significantly (*p* ≤ 0.001) higher than those of *G. krucki* and *B. mori* by 1.3 and 1.9 folds, respectively. The lowest Mg concentration was recorded in frass fertilizer produced by *B. mori*. The frass fertilizer produced by all the insect species would supply 13–34 kg Mg ha^−1^ per season if used applied fertilizer at a rate of 5 t ha^−1^ (Table 3).

There were significant differences in the concentration of total S in frass fertilizer produced by different insects (*p* ≤ 0.001) (Fig. 1f). *Hermertia illucens* produced frass fertilizer with the highest S concentration, significantly (*p* ≤ 0.001) higher than those of frass fertilizer from other insects by 1.4–3.8 folds. The concentrations of S in frass fertilizer from the *P. sinuata*, *G. bimaculatus* and *S. icipe* were significantly (*p* ≤ 0.001) higher than those of other insect frass fertilizer samples, except *H. illucens*. It was noted that the S concentration of frass fertilizer from *P. sinuata* was significantly higher than those of frass fertilizer produced by *G. bimaculatus* and *S. icipe* and *T. molitor* by 17, 29 and 53%, respectively. Also, *T. molitor* produced frass fertilizer with significantly higher S concentration than the frass fertilizer produced by *O. rhinoceros*, *S. gregaria* and *B. mori* whose frass fertilizer had the lowest S concentration. The frass fertilizer from all the insects would supply 14–35 kg S ha^−1^ per season, except *S. icipe* (Table 3).

### Concentrations of micronutrients in frass fertilizer produced by different insect species

There were significant (*p* ≤ 0.001) differences in the total concentrations of Mn, Fe, Zn, Cu, B, Na, and Al in frass fertilizer produced by different insects (Table 2). The Mn concentration ranged between 128 and 4600 mg kg^−1^, *O. rhinoceros* and *B. mori* produced frass fertilizer with the lowest and highest Mn concentrations, respectively. The concentrations of Mn in frass fertilizer produced by *O. rhinoceros* and *P. sinuata* were significantly (*p* ≤ 0.001) higher than those of frass fertilizer generated by other insects by 1.7–35 folds and 6.5–21 folds, respectively.

**Table 2:** Concentrations of micronutrients in frass fertilizers produced by different insect species.

Source of frass fertilizer	Manganese	Iron	Zinc	Copper	Boron	Sodium	Aluminium (%)
	(mg kg^−1^)						
*Hermertia illucens*	409 ± 36.2c	7803 ± 319.5c	168.0 ± 5.03d	26.9 ± 0.50b	32.6 ± 0.66d	5263.3 ± 153.0b	0.59 ± 0.020c
*Tenebrio molitor*	176 ± 14.2c	436 ± 54.1d	102.7 ± 0.33f	12.6 ± 0.07d	5.8 ± 0.45f	170.7 ± 8.1g	0.03 ± 0.003e
*Scapsipedus icipe*	431 ± 16.4c	4380 ± 196.0cd	196.0 ± 2.00ab	30.4 ± 1.07a	31.8 ± 0.19d	680.3 ± 20.4ef	0.28 ± 0.006d
*Bombyx mori*	128 ± 5.6c	887 ± 56.2d	16.1 ± 0.60g	8.9 ± 0.35e	118.0 ± 3.61a	39.8 ± 3.0g	0.07 ± 0.004e
*Gryllus bimaculatus*	359 ± 2.5c	3643 ± 84.1cd	208.0 ± 1.53a	29.9 ± 0.18ab	28.5 ± 1.12de	957.7 ± 12.3e	0.25 ± 0.003d
*Gonimbrasia krucki*	225 ± 35.0c	1825 ± 615.7d	13.8 ± 2.68g	4.3 ± 0.50f	35.3 ± 0.97d	1546.7 ± 56.1d	0.13 ± 0.033e
*Pachnoda sinuata*	2660 ± 50.3b	21,767 ± 1762.9b	195.3 ± 2.73b	28.6 ± 0.60ab	49.0 ± 1.91c	7626.7 ± 148.5a	1.77 ± 0.059b
*Schistocerca gregaria*	335 ± 18.9c	3187 ± 211.7d	117.0 ± 3.51e	17.8 ± 0.98c	23.5 ± 1.43e	566.7 ± 13.3f	0.30 ± 0.017d
*Oryctes rhinoceros*	4460 ± 484.5a	43,333 ± 2488.2a	183.0 ± 3.22c	30.6 ± 1.08a	73.2 ± 1.90b	2320.0 ± 76.4c	2.94 ± 0.027a
χ^2^ value	684.1	1470.2	5994.4	1794.4	3161.7	8883.3	11,847.0
*df*	8	8	8	8	8	8	8
*p* value	≤ 0.001	≤ 0.001	≤ 0.001	≤ 0.001	≤ 0.001	≤ 0.001	≤ 0.001

In the same column, means (± standard error) followed by the same letters are not significantly different at *p* ≤ 0.05, n = 3.

The concentration of Fe in *O. rhinoceros* frass fertilizer was 2–99 folds higher (*p* ≤ 0.001) than those of frass fertilizer produced by insects (Table 2). The concentration of Fe in *P. sinuata* frass fertilizer was significantly (*p* ≤ 0.001) higher than those of other insect frass fertilizers, except *O. rhinoceros*. The concentration of Fe in *H. illucens* frass fertilizer was 2.5, 4, 9, and 17 times higher (*p* ≤ 0.001) than those of frass fertilizer produced by *S. gregaria*, *G. krucki*, *B. mori*, and *T. molitor*, respectively. *Gryllus bimaculatus* produced frass fertilizer with 1.1–15 times higher (*p* ≤ 0.001) Zn concentration than other insects, except *S. icipe.* The concentrations of Zn in frass fertilizer produced by *T. molitor* and *G. krucki* were significantly (*p* ≤ 0.001) lower than those of frass fertilizer produced by other insects.

The concentration of Cu ranged between 4 and 31 mg kg^−1^ whereby, *G. krucki* and *O. rhinoceros* produced frass fertilizer with highest and lowest values, respectively (Table 2). The concentration of Cu in *O. rhinoceros* frass fertilizer was significantly (*p* ≤ 0.001) higher than those of frass fertilizer produced by other insects, except *P. sinuata*, *S. icipe* and *G. bimaculatus*. *Hermertia illucens* frass fertilizer had 51, 114, 202 and 526% (*p* ≤ 0.001) higher Cu concentration than the frass fertilizer produced by *S. gregaria*, *T. molitor*, *G. krucki* and *B. mori*, respectively. Furthermore, *S. gregaria* produced frass fertilizer with 1.4, 2 and 4 times higher (*p* ≤ 0.001) Cu concentration than frass fertilizer from *T. molitor*, *B. mori*, and *G. krucki*, respectively.

The concentrations of B in frass fertilizer produced by *B. mori* and *O. rhinoceros* were significantly (*p* ≤ 0.001) higher than those in frass fertilizer from other insects by 1.6–20 folds and 1.5–13 folds, respectively (Table 2). Also, *P. sinuata* produced frass fertilizer with significantly (*p* ≤ 0.001) higher B concentration than other insects, except *B. mori* and *O. rhinoceros*. The concentrations of B in frass fertilizer produced by *G. krucki*, *S. icipe* and *H. illucens* were significantly (*p* ≤ 0.001) higher than those of frass fertilizer from *S. gregaria* and *T. molitor*. *Tenebrio molitor* produced frass fertilizer with a significantly (*p* ≤ 0.001) lower concentration of B than other insects.

The concentration of Na in frass fertilizer produced by different insects is presented in Table 2. *Pachnoda sinuata* and *B. mori* produced frass fertilizer with highest and lowest Na concentration, respectively. It was noted that the concentration of Na in *P. sinuata* frass fertilizer was 1.5–192 folds higher (*p* ≤ 0.001) than those of frass fertilizer produced by other insects. *Hermertia illucens*, *O. rhinoceros* and *G. krucki* produced frass fertilizer with significantly (*p* ≤ 0.001) higher Na concentrations than other insects, except the *P. sinuata*.

The highest concentration of Al was recorded in frass fertilizer produced by the *O. rhinoceros*, significantly (*p* ≤ 0.001) higher than those of other insect frass fertilizer samples by 1.7–42 folds. Frass fertilizer from *P. sinuata* and *H. illucens* had significantly (*p* ≤ 0.001) higher Al concentrations than other insects’ frass fertilizer, except *O. rhinoceros*. Also, the concentrations of Al in frass fertilizer from *S. gregaria*, *G. bimaculatus and S. icipe* were significantly (*p* ≤ 0.001) higher than those of frass fertilizer produced by *G. krucki*, *B. mori*, and *T. molitor* which had the lowest concentration.

At the application rate of 5 t ha^−1^, frass fertilizer from eight insect species would supply less than 1 kg ha^−1^ of Cu, B and Zn. Out of the nine insect species, only *G. bimaculatus* frass fertilizer would supply at least 1 kg Zn ha^−1^ (Table 3). Apart from *P. sinuata* and *O. rhinoceros*, frass fertilizer from the other insects would supply less than 5 kg of Mn ha^−1^. Frass fertilizer from the various insects would supply 16–217 kg Fe ha^−1^ if applied at a rate of 5 t ha^−1^, except for *T. molitor*, *B. mori* and *G. krucki* (Table 3).

**Table 3:** Nutrients in frass fertilizers if applied at the rate of 5 t ha^−1^ for crop production.

Source of frass fertilizer	Nitrogen	Phosphorus	Potassium	Calcium	Magnesium	Sulphur	Zinc	Manganese	Iron	Copper	Boron
	(kg ha^−1^)										
*Hermertia illucens*	147.5 ± 4.1	63.2 ± 2.7	203.7 ± 4.2	52.7 ± 2.2	26.2 ± 0.93	34.5 ± 0.76	0.84 ± 0.03	2.1 ± 0.18	39.0 ± 1.60	0.14 ± 0.00	0.16 ± 0.00
*Tenebrio molitor*	124.3 ± 2.3	69.8 ± 2.6	104.8 ± 1.5	15.8 ± 0.6	28.5 ± 0.87	15.8 ± 0.17	0.51 ± 0.00	0.9 ± 0.07d	2.2 ± 0.27	0.06 ± 0.00	0.03 ± 0.00
*Scapsipedus icipe*	120.7 ± 1.2	74.2 ± 1.7	122.7 ± 0.7	139.8 ± 4.5	27.0 ± 0.58	19.0 ± 0.28	0.98 ± 0.01	2.2 ± 0.08	21.9 ± 0.98	0.15 ± 0.01	0.16 ± 0.00
*Bombyx mori*	117.2 ± 3.3	8.5 ± 0.3	86.5 ± 0.9	173.7 ± 7.4	12.7 ± 0.33	9.2 ± 0.17	0.08 ± 0.00	0.6 ± 0.03	4.4 ± 0.28	0.04 ± 0.00	0.59 ± 0.02
*Gryllus bimaculatus*	114.5 ± 2.1	76.7 ± 1.6	103.7 ± 1.4	147.3 ± 5.8	24.5 ± 0.29	20.8 ± 0.17	1.04 ± 0.01	1.8 ± 0.01	18.2 ± 0.42	0.15 ± 0.00	0.14 ± 0.01
*Gonimbrasia krucki*	107.0 ± 1.3	9.8 ± 1.1	70.3 ± 2.9	94.7 ± 2.5	17.3 ± 0.17	14.7 ± 0.17	0.07 ± 0.01	1.1 ± 0.18	9.1 ± 3.08	0.02 ± 0.00	0.18 ± 0.01
*Pachnoda sinuata*	106.8 ± 3.3	52.2 ± 0.7	151.2 ± 0.4	121.2 ± 2.1	34.2 ± 0.44	24.3 ± 0.17	0.98 ± 0.01	13.3 ± 0.25	108.5 ± 8.81	0.14 ± 0.00	0.25 ± 0.01
*Schistocerca gregaria*	103.3 ± 0.7	36.5 ± 1.0	175.3 ± 8.0	62.7 ± 4.9	26.3 ± 0.73	13.7 ± 0.44	0.59 ± 0.02	1.7 ± 0.10	15.9 ± 1.05	0.09 ± 0.01	0.12 ± 0.01
*Oryctes rhinoceros*	82.8 ± 2.2	21.5 ± 0.5	82.0 ± 0.3	108.5 ± 1.5	23.5 ± 0.00	14.0 ± 0.29	0.92 ± 0.02	22.3 ± 2.42	216.7 ± 2.44	0.15 ± 0.01	0.37 ± 0.01

Mean (± standard error), n = 3.

### Fertilizing index of frass fertilizer produced by different insect species

The fertilizing indices of frass fertilizer produced by different insects ranged between 3.9 and 4.8, whereby, *G. krucki* and *H. illucens* produced frass fertilizer with the lowest and highest values, respectively. The fertilizing indices of frass fertilizer produced by other insects were than those of *B. mori* and *G. krucki* frass fertilizer.

### Maturity status of frass fertilizer produced by different insect species

The pH and electrical conductivity (EC) of frass fertilizer produced by different insects varied significantly (*p* ≤ 0.001) (Table 4). The pH ranged between 4.6 and 8.3, whereby *G. krucki* and *P. sinuata* produced frass fertilizer with lowest and highest pH values, respectively. The pH of frass fertilizer produced by *P. sinuata* and *B. mori* were significantly (*p* ≤ 0.001) higher than those of frass fertilizer from other insects. Furthermore, the *O. rhinoceros*, *H. illucens* and *S. icipe* produced frass fertilizer with significantly (*p* ≤ 0.001) higher pH values than the *T. molitor*, *G. bimaculatus, S. gregaria* and *G. krucki*.

**Table 4:** Compost maturity indices of frass fertilizer produced by different edible insect species.

Source of frass fertilizer	pH	Electrical conductivity (mS cm^−1^)	Ammonium/nitrate ratio	C/N ratio	Seed germination rate	Gemination index
					(%)	
*Hermertia illucens*	7.5 ± 0.09c	16.0 ± 1.58b	3.9 ± 3.83b	13.2 ± 0.39b	93.3 ± 3.3a	267.1 ± 91.7a
*Tenebrio molitor*	6.5 ± 0.05e	6.7 ± 0.63f	0.006 ± 0.00b	20.0 ± 0.35ab	56.7 ± 14.5c	27.9 ± 6.2e
*Scapsipedus icipe*	7.4 ± 0.02c	11.5 ± 0.07cde	0.005 ± 0.00b	17.4 ± 0.35ab	63.3 ± 3.3bc	31.2 ± 1.6e
*Bombyx mori*	8.1 ± 0.02a	10.0 ± 0.69def	1.00 ± 0.00b	19.1 ± 4.94ab	60.0 ± 5.8bc	29.5 ± 2.8e
*Gryllus bimaculatus*	6.6 ± 0.05e	9.0 ± 0.34ef	0.12 ± 0.00b	18.6 ± 0.32ab	76.7 ± 3.3abc	37.8 ± 1.6de
*Gonimbrasia krucki*	4.6 ± 0.04f	15.5 ± 0.23b	79.7 ± 10.1a	22.9 ± 0.14a	86.7 ± 3.3ab	47.8 ± 6.2cd
*Pachnoda sinuata*	8.3 ± 0.03a	25.1 ± 0.18a	1.58 ± 0.009b	14.6 ± 0.56b	96.7 ± 3.3a	66.1 ± 8.5bc
*Schistocerca gregaria*	6.9 ± 0.03d	13.9 ± 0.16bc	0.003 ± 0.00b	19.5 ± 0.47ab	23.3 ± 6.7d	5.8 ± 1.6f
*Oryctes rhinoceros*	7.9 ± 0.02b	13.2 ± 0.25bcd	0.15 ± 0.05b	14.6 ± 0.85b	93.3 ± 6.7a	73.9 ± 12.4b
χ^2^-value	5195.2	330.4	424.4	26.5	105.9	1602.0
*df*	8	8	8	8	8	8
*p* value	≤ 0.001	≤ 0.001	≤ 0.001	≤ 0.001	≤ 0.001	≤ 0.001

C/N ratio = ratio of total organic carbon to total nitrogen. In the same column, means (± standard error) followed by the same letters are not significantly different at *p* ≤ 0.05, n = 3.

All insects produced frass fertilizer with EC values greater than 6 mS cm^−1^ (Table 4). The highest EC was recorded in frass fertilizer produced by *O. rhinoceros*, significantly (*p* ≤ 0.001) higher than other frass fertilizer samples by 1.7–3.8 folds. Also, the EC of frass fertilizer from *H. illucens* and *G. krucki* was significantly (*p* ≤ 0.001) higher than those of *S. icipe, B. mori*, *G. bimaculatus* and *T. molitor*.

The ratios of ammonium to nitrate, and total organic carbon to total nitrogen (C/N ratio) also varied significantly among frass fertilizer samples (*p* ≤ 0.001) (Table 4). The ratios of ammonium to nitrates ranged between 0.01 and 79.5, frass fertilizer from *S. gregaria* and *G. krucki* had the highest and lowest values, respectively. Frass fertilizer from *T. molitor*, *S. icipe, B. mori*, *G. bimaculatus, S. gregaria* and *O. rhinoceros* achieved ammonium to nitrate ratios of ≤ 1. The C/N ratios of 13–23 were observed during experiments, whereby the *H. illucens* and *G. krucki* produced frass fertilizer with the lowest and highest C/N ratios, respectively. *Gonimbrasia krucki* frass fertilizer had significantly (*p* ≤ 0.001) higher C/N ratio than frass fertilizer fertilizers produced by *P. sinuata, O. rhinoceros* and *H. illucens*.

There were significant differences (*p* ≤ 0.001) in seed germination rate and germination indices of seeds exposed to frass fertilizer produced by various insect species (Table 4). Frass fertilizer from *P. sinuata* achieved the highest (97%) seed germination rate, 4 folds higher (*p* ≤ 0.001) than the value achieved using *S. gregaria* frass fertilizer. Furthermore, the seed germination rates of frass fertilizer produced by *P. sinuata*, *O. rhinoceros* and *H. illucens* were significantly (*p* ≤ 0.001) higher than that of the *S. gregaria*, *B. mori*, *S. icipe* and *T. molitor*. The seed germination rate of *G. krucki* frass fertilizer was significantly (*p* ≤ 0.001) higher than those of frass fertilizer from *S. gregaria* and *T. molitor* (3.7 and 1.5 folds, respectively).

*Hermertia illucens* frass fertilizer achieved the highest germination index (267%), significantly (*p* ≤ 0.001) higher than the values obtained using other frass fertilizer samples by 3.7–46 folds (Table 4). The lowest germination index (6%) was obtained using frass fertilizer produced by *S. gregaria*. The germination index of *O. rhinoceros* frass fertilizer was significantly (*p* ≤ 0.001) higher than those of frass fertilizer produced by *S. gregaria*, *T. molitor*, *B. mori*, *S. icipe, G. bimaculatus*, and *G. krucki* by 12.7, 2.6, 2.5, 2.4, 2.0 and 1.6 folds, respectively. *Pachnoda sinuata* frass fertilizer achieved significantly (*p* ≤ 0.001) higher germination index than the other insect frass fertilizer samples, except for *H. illucens*, *O. rhinoceros* and *G. krucki* (Table 4).

### Multivariate analysis of compost quality parameters

The principal component analysis (PCA) revealed that nutrient concentration, fertilizing index, and compost maturity were highly affected by the species of insects used to produce the frass fertilizer (Fig. 3). The first two components of the PCA accounted for 62% of the total variance whereby, PC1 accounted for 37% and PC2 accounted for 25%. It was noted that total phosphorus, potassium, sulphur, magnesium zinc, copper sodium, germination index and fertilizing were positively correlated, but negatively correlated with ammonium and ammonium to nitrate ratio (Fig. 3). The pH, electrical conductivity, germination rate, aluminium, iron, manganese, and nitrates were also highly correlated, but negatively correlated with total organic carbon. Total nitrogen, moisture content and total organic carbon were also highly correlated.

## Discussion

### Nutrient levels of frass fertilizer generated by different insect species and potential for fertilizer use

**Figure 2:**
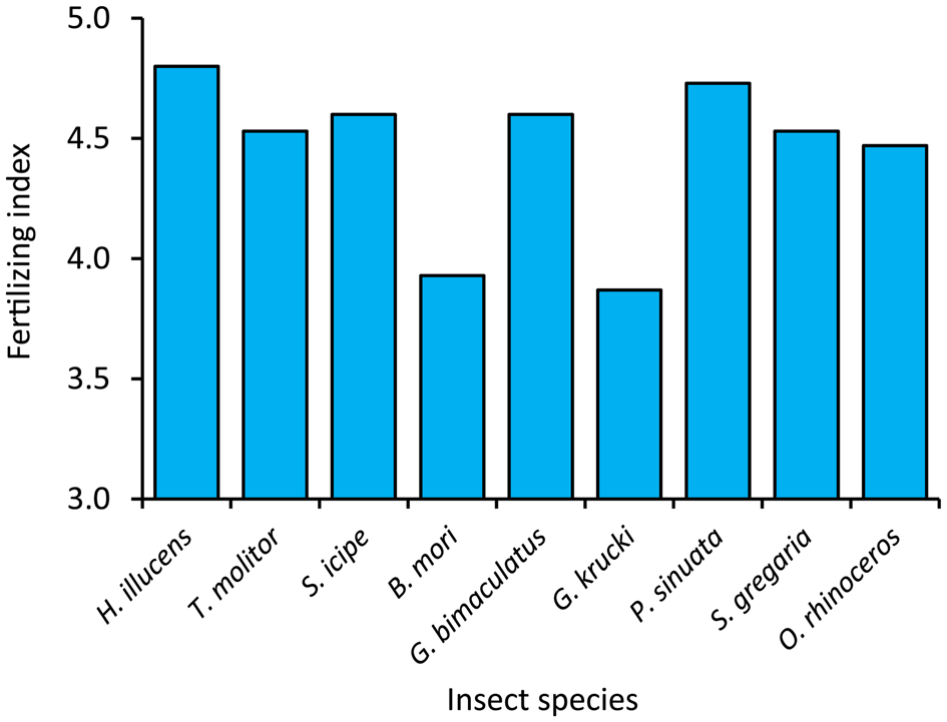
Fertilizing indices of frass fertilizer produced by different edible insect species, n = 3.

Analysis of frass fertilizer from the nine insect species indicates high potential for use as high-quality and affordable alternative source of fertilizer compared to the scarce, costly and poor-quality organic fertilizers present in most regions of SSA^14,17^. The fertilizing index values of above three achieved by frass fertilizer produced by all the nine insect species (Fig. 2) also confirm the high suitability of insect frass fertilizer as a sustainable source of plant nutrients and quality fertilizer input for sustainable soil health management^45^.

The high quantities of nitrogen, phosphorus, potassium, and micronutrients that would be supplied for crop production per season show that insect frass fertilizers could be relied on exclusively to cater for the nutrient demands of the key crop food and cash crop grown in SSA. For example, previous studies have reported higher yields and nutritional quality of maize, tomatoes, kales, French beans, cow peas, chilli pepper, shallots and barley grown using *H. illucens* frass fertilizer and meal worm frass fertilizer compared to conventional fertilizers^27,28,42,52,53^. Furthermore, soil amendment with frass fertilizer from *H. illucens* and *T. molitor* was found to suppress soil borne pathogens, stimulate soil microbial activity, reduce soil acidity and salinity, improve nitrogen mineralization, and increase availability of nutrients in the soil^29–31,33,53^, thus improving the soil quality for plant growth. It is therefore anticipated that adoption of insect fertilizer would contribute to improving food security by in SSA reversing the worrying trends in soil degradation, nutrient mining and declining crop productivity^1,8,14^.

The differences in nutrient concentrations and nutrient supply potential observed using frass fertilizer generated by different insect species highlights the variations in bioconversion and nutrient recycling efficiencies^23^. This could be largely attributed to differences in the nutritional quality of substrates used in rearing the various insect species^39,40,54,55^. For example, the higher concentration nitrogen, phosphorus and potassium in frass fertilizer produced by *H. illucens*, *T. molitor*, *G. bimaculatus* and *S. icipe* could be largely attributed to the high nutrient levels in the brewery spent grain, potato peels, wheat bran, soy bean and other substrates used to rear these insects^26,56,57^. In comparison however, *G. krucki* and *B. mori* were exclusively fed on tree branches with lower nutritional quality, thus producing frass fertilizer with less fertilizer potential. Nevertheless, the fertilizing indices of frass fertilizer produced by the two insect species were also above three, indicating potential for use as organic fertilizers^45^. There is inadequate research on the agronomic performance of most frass fertilizers assessed during the study. Therefore, field application of frass fertilizers would require prior agronomic studies to establish the optimal soil amendment rates for high nutrient release and synchrony for crop uptake, nutrient use efficiency, and crop yield and nutritional quality.

We found that the concentrations N, P, K, Ca, Mg, and S (> 1%) and those of Mn, Zn, Cu, Fe and B (> 0.1%) in frass fertilizers produced by all the nine insect species were within the recommended standards according to the Kenya Bureau of Standards guidelines for optimal commercial organic fertilizer^58^. Also, the nutrient concentrations in the various insect frass fertilizers meet the required international standards and guidelines for quality organic fertilizers in the United States, Canada and the European Union^59^.

It should be noted that the waste degradation efficiencies and composting periods of insect species differ, and thus the quantity of frass fertilizer available. For example, the *H. illucens* larvae have a high waste degradation efficiency (55–80%)^60,61^ and require a short bioconversion time, and thus could produce higher amounts of organic fertilizer than other insect species. On the other hand, the *T. molitor*, *P. sinuata* and *O. rhinoceros* take 4–6 weeks to convert organic waste into frass fertilizer, compared to 2 weeks for *H. illucens* larvae^26^, while *S. gregaria* and crickets can produce frass fertilizer daily but in less quantities compared to *H. illucens* larvae.

### Maturity status of frass fertilizer generated by different insect species

Compost maturity refers to the degree of completeness of composting and absence of phytotoxic compounds and plant or animal pathogens that could negatively affect seed germination, plant growth and soil health^46,47^. In other words, compost maturity indicates the suitability of compost for field application as a fertilizer, using biological, physical and chemical indicators^62–64^. The values of C/N ratio (13–24) and ammonium concentration (0.01–56 mg kg^−1^) achieved during the study were within the ranges recommended by Goyal et al.^63^ (< 25) and Bernal et al.^62^ (< 400 mg kg^−1^), respectively, for quality compost production. Apart from *G. krucki* frass fertilizer, the pH of other insect frass fertilizer samples was within the acceptable range (6–8) for mature compost^46^.

The ratios of ammonium/nitrate of frass fertilizer produced by *T. molitor*, *S. icipe*, *G. bimaculatus*, *P. sinuata*, *S. gregaria* and *O. rhinoceros* were within the critical value (< 0.16) recommended by Bernal et al.^62^ for mature compost. On the other hand, the ratios of ammonium/nitrate recorded in frass fertilizer from *H. illucens*, *B. mori* and *G. krucki* (1–80) are comparable to the values reported by Guo et al.^65^ (1–60) and Beesigamukama et al.^26^ (0.3–34) for mature compost. This implies that the frass fertilizer produced by all the nine insect species can release adequate of quantities of nutrients once applied in the soil, thus high potential for improving soil and crop productivity. It is important to note that for all the frass fertilizer samples, the values of moisture content, ammonium concentration (Table 1), C/N and pH (Table 4), obtained were within the ranges recommended for mature and stable compost in Kenya^58^, the United States, Canada and European Union^59^.

The high seed germination rate (> 90%) and germination index (267%) achieved using *H. illucens* frass fertilizer indicate absence of phytotoxicity (Table 4), thus capacity to support optimal plant growth^48^. In comparison however, the germination index values of < 50% obtained using frass fertilizer from *T. molitor*, *S. icipe*, *B. mori*, *G. krucki* and *S. gregaria* indicate high phytotoxicity, thus low capacity to support crop growth without further treatment^49^. The germination index values of 50–80% achieved using frass fertilizer generated by *P. sinuata* and *O. rhinoceros* indicate moderate phytotoxicity and minimal suitability for crop production. Composts with high and moderate phytotoxicity have been reported to impair seed germination and radical elongation^48,49^.

**Figure 3:**
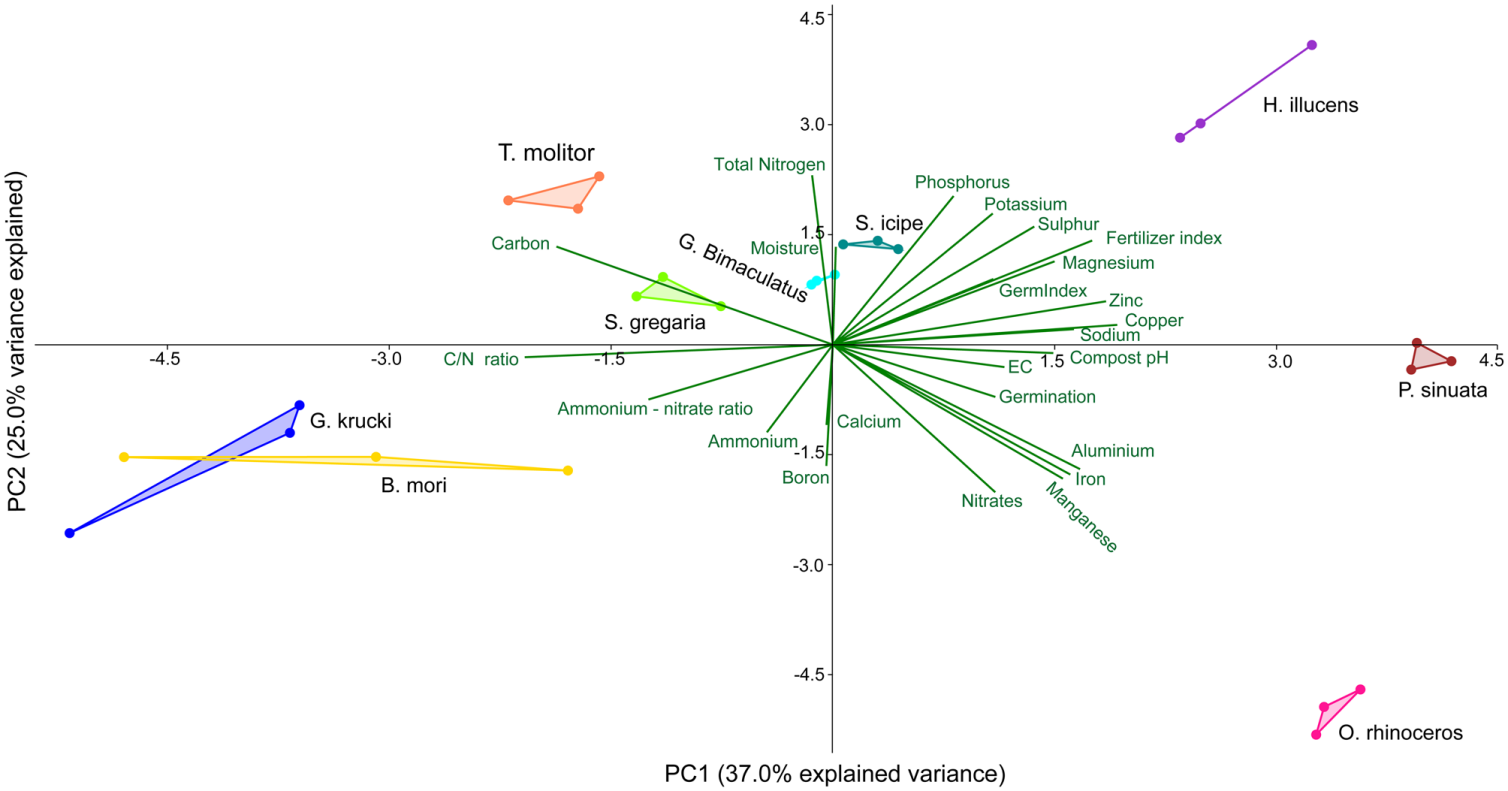
Biplot graphs based on the principal component (PC) analysis of parameters that measure the quality of frass fertilizer composts produced by different edible insect species (n = 3).

The high and moderate levels of phytotoxicity observed in the above insect frass fertilizers could be attributed to the high salt (cation) concentration (Table 2) and high electrical conductivity (Table 3, Fig. 3)^49,66^. The values of electrical conductivity recorded in all frass fertilizers were above the allowable limit of < 4 mS cm^−1^^67^. The low germination index and high electrical conductivity observed in all frass fertilizers except for *H. illucens* frass fertilizer, highlight the need for further composting and leaching to eliminate all the phytotoxic substances and excess salts, respectively. Future studies will be necessary to determine the time required to achieve full compost maturity and stability of frass fertilizer produced by the different insect species to improve suitability for field application.

## Conclusion

Here, we present the first comparative report on the quality of frass fertilizer generated by nine edible insect species to offer specific guidelines on their effective use for improved soil health and crop productivity. The high nutrient levels, fertilizing indices and potential nutrient supply capacities of various frass fertilizer products indicate their suitability as sustainable alternative sources of plant nutrients. It is anticipated that the availability and adoption of insect frass fertilizers would significantly reduce overreliance on the unaffordable commercial mineral fertilizers as well as poor quality organic fertilizers. Frass fertilizer products from the *H. illucens* and the two cricket species (*G. bimaculatus* and *S. icipe*) were the best in terms of nutrient concentration, and potential supply capability. However, the frass fertilizer from all the insects, except *H. illucens* would require further composting to improve their maturity and stability. Further agronomic studies to establish the optimal amendment rates of the various frass fertilizers to ensure high nutrient release and synchrony for crop uptake, improved yield, and nutritional quality of food crops are crucial.

## Materials and methods

### Source of frass fertilizer compost samples

The frass fertilizer samples were sourced from insect colonies at Animal Rearing and Containment Unit (ARCU) at *icipe* (01° 13′ 25.3″ S, 36°53′ 49.2″ E, 1600 m asl), Nairobi, Kenya. *Hermertia illucens* frass fertilizer was obtained by feeding the larvae on a mixture of Irish potato peels and brewery spent grain for two weeks following procedures described by Beesigamukama et al.^26^. *Schistocerca gregaria* frass fertilizer was obtained from a colony of locusts fed on diet consisting of wheat and barley seedlings and wheat bran in a room maintained at 30 ± 4 °C, 40–50% relative humidity and a photoperiod of 12:12 L:D^68^.

The cricket frass fertilizer samples were obtained by feeding neonates on a diet consisting wheat bran, soy bean, sweet potato vines and weeds for a period of 2 months and 3 months for *G. bimaculatus* and *S. icipe*, respectively, following procedures described by Magara et al.^56^. Frass fertilizer sample of the *P. sinuata* and *O. rhinoceros* were obtained by feeding them on fresh cattle dung from a dairy farm for a period of 4–5 weeks. The pelletized faeces of the beetles were collected and stored for further analysis and utilization.

*Tenebrio molitor* frass fertilizer was obtained by feeding mealworms on wheat bran and chayote (*Sechium edule*) for a period of five weeks, following procedures described by Thévenot et al.^69^. *Bombyx mori* frass fertilizer was obtained by feeding silkworms on the leaves of mulberry tree (*Morus spp.*) for a period of six weeks, following the procedures described by Hailu^70^ and Nguku et al.^71^. For *G. krucki*, the frass fertilizer was obtained by feeding them on the Brazilian pepper tree leaves (*Schinus terebinthifolia* Raddi) for a period of 5 weeks. The frass fertilizer samples collected from various insect species were air-dried for five days pending laboratory analysis. Frass fertilizer products from crickets, beetles, *S. gregaria*, *B. mori* and *G. krucki* were in pelletized form, while that of the *H. illucens* and *T. molitor* were in powder form.

### Determination of frass fertilizer quality

The fertilizer quality of the frass fertilizer generated by the different edible insects was assessed by determining the concentrations of macro—(total organic carbon [C], nitrogen [N], phosphorus [P] and potassium [K]), secondary—(calcium [Ca], magnesium [Mg] and sulphur [S]), and micro-nutrients (manganese [Mn], iron [Fe], zinc [Zn], copper [Cu], boron [B], sodium [Na] and aluminium [Al]) using standard laboratory methods described in “Concentrations of micronutrients in frass fertilizer produced by different insect species” section. Nutrient concentrations were determined using air-dried frass fertilizer samples. Furthermore, the C/N ratio and concentrations of C, N, P and K were used to determine the fertilizing index of frass fertilizers for soil and crop productivity using Eq. (1)^45^. On 5.0-point scale, only frass fertilizers with a fertilizing index of greater than 3.0 were considered suitable for use as organic fertilizer^45^

1 $$ Fertilizing\;index = \frac{{\sum\nolimits_{n}^{i = 1} {S_{i} W_{i} } }}{{\sum\nolimits_{n}^{i = n} {W_{i} } }} $$

where *S*_*i*_ represents the score value of analytical data. The *S*_*i*_ scale ranges from 1 to 5; per parameter, a score of 1 represented the lowest concentration/value while 5 represented the highest. *W*_*i*_ represents the weighing factor of the *i*th fertility parameter. The scores of *W*_*i*_ were based on scientific knowledge on the roles of the nutrients and parameters in improving soil productivity. Consequently, *W*_*i*_ scores of 5, 3, 3, 1 and 3 were used for C, N, P, K and C/N ratio, respectively, according to Saha et al.^45^.

The concentrations of nutrients in different frass fertilizers were used to determine the amount of nutrients (N, P, K, Ca, Mg, S, Mn, Zn, Cu, Fe and B) that would be released for crop production per season per hectare (kg ha^−1^). The organic fertilizer application rate of 5 t ha^−1^ which is recommended in study country (Kenya) was used in the calculations (Eq. 2)^51,72–75^

2 $$ Amount\;of\;nutrients\left( {{\text{kg}}\;{\text{ha}}^{ - 1} } \right) = \frac{{\left[ {Quantity\;of\;nutrients\; in\;100\;{\text{kg}}\;of\;frass \left( {{\text{kg}}} \right) \times 5000} \right]}}{100} $$

where 5000 represents organic fertilizer application rate of 5000 kg ha^−1^ (dry weight).

Compost maturity and stability were assessed to determine if the frass fertilizer generated by various insects species were ready for use as organic fertilizers (freedom from compounds that could negatively affect seed germination and/or plant growth)^46^. Compost maturity and stability were determined using pH (6–8), ammonium concentration (< 400 mg kg^−1^), ammonium to nitrate ratio (< 0.16)^46,62^, electrical conductivity (< 4 mS cm^−1^)^67^ and C/N ratio (< 20)^63^, following the procedures described in “Concentrations of micronutrients in frass fertilizer produced by different insect species” section. Compost phytotoxicity tests were performed by determining seed germination index (> 80%), following standard procedures^48,49^. All tests compost maturity and stability were carried out using fresh frass fertilizer samples.

### Laboratory analysis methods

Laboratory-based analysis of frass fertilizer pH and EC was carried out using aqueous extracts of 1:10 (weight/volume) compost to distilled water. The contents were then shaken for 30 min at 180 revolutions min^−1^ on an orbital and linear shaker (MI0103002, Foure’s scientific, China). The pH and EC were then read directly using a pH (AD1000, Adwa, Romania) and EC meter (AVI, Labtech, India), respectively^76^. The nitrate and ammonium were extracted from frass fertilizer using 0.5 M potassium sulphate at a ratio of 1:10 (weight/volume). Thereafter, the entire content of compost-potassium sulphate mixture was shaken for 1 h using an orbital and linear shaker (KOS–3333/KCS–3333, MRC, UK) as described above. The solution was later filtered through a Whatman No. 1 filter paper and the filtrate was used for further analyses. Furthermore, the nitrate and ammonium concentrations were determined by colorimetric methods as described by Okalebo et al.^76^.

Total organic carbon was determined using the wet oxidation method^77^ while total N, P, K, Ca, Mg, Mn, Fe, Zn, Cu, B, Na and Al were extracted using acid digestion method^76^. A sample weight of 0.3 g was used in the digestion. The nutrients were extracted using 10 ml of digestion mixture made by dissolving 0.42 g of selenium powder and 14 g of lithium sulphate in 350 ml of 30% hydrogen peroxide and 420 ml of concentrated sulphuric acid. The digestion process was carried out in 250 ml digestion tubes at temperatures of 360 °C for three hours to obtain a colourless solution (digestate) that was later used during the analysis of the different nutrients. Total N in the digestate was determined using the Kjeldahl distillation and titration method^78^. Total phosphorus was determined using the Ultraviolet–visible (UV–Vis) spectroscopy method^76^ by complexing the digestate with the solution containing sulphuric acid, ammonium molybdate, antimony potassium tartrate and ascorbic acid. Total K and Na were determined using flame photometry^76^.

The total concentrations of Ca, Mg, Mn, Fe, Zn and Cu in the digestates were determined using atomic absorption spectrometry (AAS) (iCE 3300 AA system, Thermo scientific, China) and element-specific wavelengths of 422.7, 285.2, 248.3, 248.3, 213.9, 324.7 nm, respectively^76^. The total concentrations of B and Al were determined using inductively coupled plasma-atomic emission spectrometry (249.77 nm) and dithionite–citrate method followed by AAS, respectively^79^.

The concentration of sulphur in frass fertilizer samples was extracted by heating 0.5 g in a muffle furnace at 450 °C for 2 h^76^. After cooling, 5 ml of 6 N HCl were added to the residue and the mixture was digested at 150 °C using for 25 min. After cooling, contents were transferred quantitatively to 100 ml volumetric flask, and topped up to the mark with distilled water. The extracts were then filtered through the extract through Whatman No. 40 filter paper. The total concentration of S in the filtrate was determined using the turbidimetric method at 420 nm, following procedures described in Okalebo et al.^76^.

### Phytotoxicity test on frass fertilizer extracts

Seed germination index was determined by growing cabbage seeds in the various insect frass fertilizer extracts. Ten cabbage seeds were randomly selected and placed on petri dishes lined with filter paper moistened with 10 ml of 10% insect frass fertilizer extracts for 96 h at 25 °C in a dark chamber. The same procedure was repeated using distilled water as a positive control. After 96 h, germinated seeds were counted, and their radicle lengths measured. Germination index (GI) was calculated using Eq. (3)^48^. Frass fertilizer samples with GI values below 50% were considered highly phytotoxic, while values between 50 and 80% were moderately phytotoxic; and values above 80% indicated no phytotoxicity^48,49^

3 $$ GI\left( \% \right) = \frac{RSG\left( \% \right) \times RRG\left( \% \right)}{{100}} $$

where *RSG* (%) represents the relative seed germination calculated as:

$$ RSG = \frac{number\;of\;seeds\;germinated\;in\;frass\;extract}{{number\;of\;seeds\;germinated\;in\;control\left( {distilled\;water} \right)}} \times 100. $$

*RRG* (%) represents the relative root growth calculated as:

$$ RRG = \frac{mean\;root\;length\;in\;frass\;extract}{{mean\;root\;length\;in\;control\left( {distilled\;water} \right)}} \times 100. $$

### Data analysis

Before analysis, all data were checked for normality using the Shapiro–Wilk test. Normally distributed data was analysed using one-way analysis of variances, while data that was not normally distributed was analysed using generalised liner model (GLM) followed by analysis of deviances. Computation of least squares means was done using “lsmeans” package, followed by mean separation using adjusted Tukey’s method at *p* ≤ 0.05, implemented using “cld” function from the “multicompView” package. Principal component analysis was performed using the “prcomp” function from the “ggbiplot” package to examine the relationship among the frass fertilizer quality parameters. All the statistical analyses were conducted using R software version 4.0.3^80^.

## Data Availability

All relevant data are presented in the paper.
